# Strategies for Accessing *cis*-1-Amino-2-Indanol

**DOI:** 10.3390/molecules29112442

**Published:** 2024-05-22

**Authors:** Inès Mendas, Stéphane Gastaldi, Jean-Simon Suppo

**Affiliations:** Aix Marseille Univ, CNRS, ICR, Marseille, France

**Keywords:** *cis*-1-amino-2-indanol, Ritter reaction, intramolecular cyclization, enantioselective reaction, enzymatic resolution, C-H activation, chemical resolution, epimerization

## Abstract

*cis*-1-amino-2-indanol is an important building block in many areas of chemistry. Indeed, this molecule is currently used as skeleton in many ligands (BOX, PyBOX…), catalysts and chiral auxiliaries. Moreover, it has been incorporated in numerous bioactive structures. The major issues during its synthesis are the control of *cis*-selectivity, for which various strategies have been devised, and the enantioselectivity of the reaction. This review highlights the various methodologies implemented over the last few decades to access *cis*-1-amino-2-indanol in racemic and enantioselective manners. In addition, the various substitution patterns on the aromatic ring and their preparations are listed.

## 1. Introduction

**Scheme 1:**
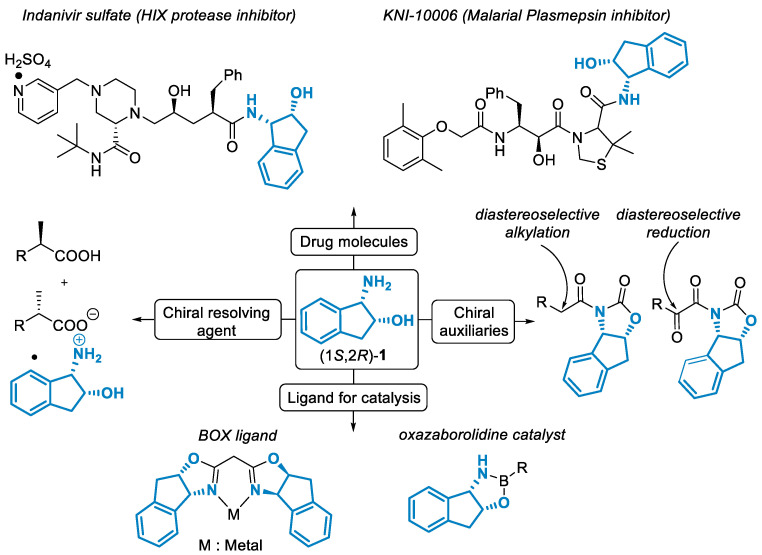
*cis*-1-amino-2-indanol structure and examples of applications.

1-Amino-2-alcohols are crucial building blocks in many fields, including medicinal and organic chemistry. Consequently, both their synthesis and the development of new methodologies to obtain them constitute an important area of organic chemistry [1,2,3,4]. More particularly, *cis*-1-amino-2-indanol (**1**) plays a central role in organic synthesis as a ligand or chiral auxiliary due to its rigid cyclic skeleton (Scheme 1). This structure is the key moiety of BOX and PyBOX ligands widely used in asymmetric catalysis [5,6,7]. Notably, oxazaborilidine catalysts derived from *cis*-1-amino-2-indanol are often more efficient than other chiral 1-amino-2-alcohol structures in the enantioselective reduction in carbonyl compounds [8]. In addition, *cis*-1-amino-2-indanol is an important derivative when used as a chiral auxiliary in several asymmetric transformations, such as diastereoselective enolate alkylation or diastereoselective reduction [9]. This compound, like other chiral amines, also has applications in the resolution of racemic carboxylic acid bearing a chiral carbon at position α [9]. It is also an interesting substructure for drug design. It is present in Indinavir sulfate (Crixivan^®^), an HIV protease inhibitor for the treatment of acquired immunodeficiency syndrome (AIDS) developed by Merck (Rahway, NJ, USA) [10,11], or in KNI-10006 for anti-malarial treatment [12].

**Scheme 2:**
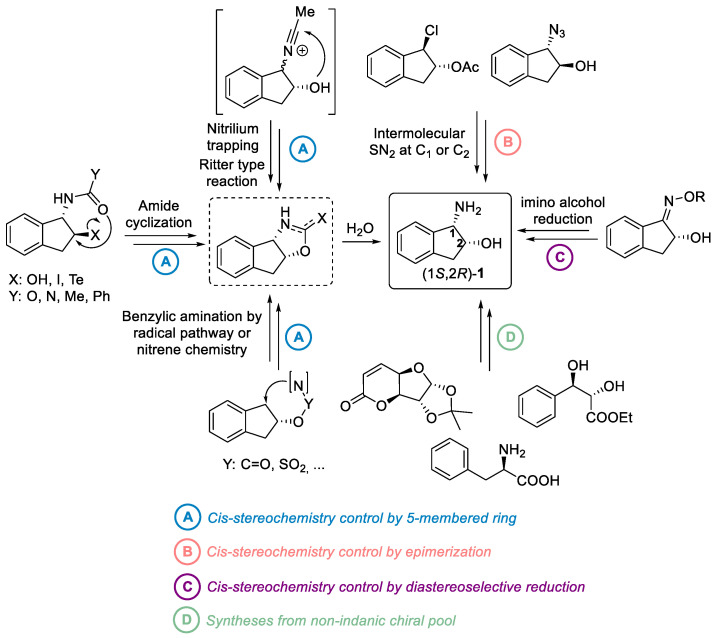
Main strategies developed for the synthesis of *cis*-1-amino-2-indanol.

Due to its wide range of applications, the synthesis of *cis*-1-amino-2-indanol has attracted the interest of numerous academic and industrial researchers, leading to the development of various synthetic pathways (Scheme 2) [9,13]. Most of these strategies involve indane skeleton as the starting point, but total syntheses from other building blocks have also been reported. Whatever the starting material, the same synthetic difficulties have to be overcome. One of the first points to control is the selective introduction of nitrogen and oxygen atoms in the -C1 and C2 positions, respectively. Afterward, the main challenge is to control the *cis* relationship between the oxygen and nitrogen atoms and to carry out the process enantioselectively.

From the indane skeleton, the main strategy for obtaining the *cis* relationship between nitrogen and oxygen atoms involves the key intramolecular formation of a *cis*-5-membered ring (Scheme 2, (A)). This approach requires a final hydrolysis step to deliver the desired *cis*-1-amino-2-indanol. Alternative methodologies have been developed relying on the epimerization of C_1_ or C_2_ centers, or diastereoselective reduction (Scheme 2, (B) and (C)). Interestingly, other syntheses have been developed from non-indanic precursors, allowing the use of the chiral pool to introduce the right configurations (Scheme 2, (D)).

## 2. Syntheses from Indane Skeleton

### 2.1. cis Stereochemistry Controlled by Intramolecular Formation of 5-Membered Ring

#### 2.1.1. Nitrilium Trapping—Ritter Type Reaction

**Scheme 3:**
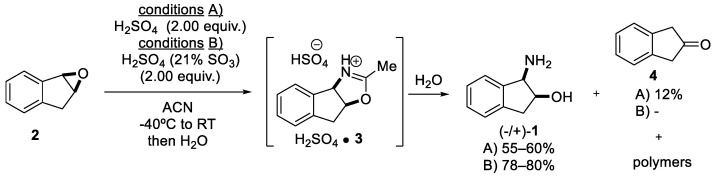
Ritter type reaction from indene oxide.

Ritter type reaction on indene oxide is a practical route for the synthesis of enantiopure *cis*-1-amino-2-indanol. This strategy was reported by Senanayake in 1995, first starting from indene oxide (**2**) prepared via the reaction of indene with hydrogen peroxide. This allowed the isolation of *cis*-1-amino-2-indanol (−/+)-**1** under acid conditions after hydrolysis of oxazoline intermediate **3** (Scheme 3) [14]. The first attempts using 2.0 equivalent of 97% sulfuric acid in acetonitrile from −40 °C to RT gave the desired compound with 55–60% yield. Interestingly, when the reaction was performed with fuming sulfuric acid (21% SO_3_), the formation of indanone **4** was suppressed, and the yield improved to 78–80%.

**Scheme 4:**
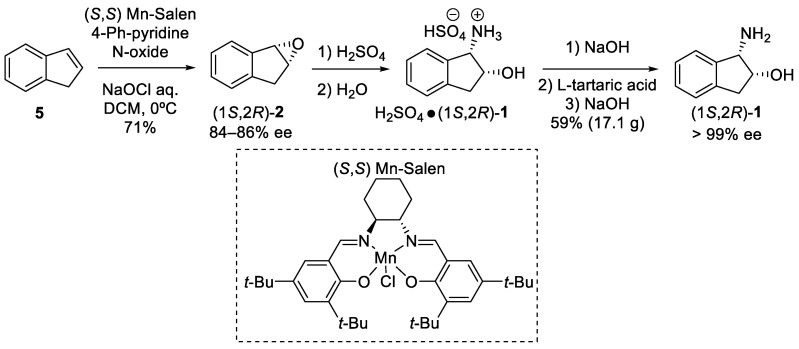
Enantioselective synthesis of (1*S*,2*R*)-1-amino-2-indanol via the Ritter process.

This strategy was then optimized, and a larger scale synthesis was proposed, enabling the attainment of 17.1 g of enantiopure (1*S*,2*R*)-**1** starting from enantioenriched indene oxide (1*S*,2*R*)-**2** obtained via Jacobsen enantioselective epoxidation of indene (**5**) (Scheme 4) [15]. After applying typical sequence Ritter reaction and hydrolysis, the resulting sulfuric acid salt (1*S*,2*R*)-**1** was engaged in an enantioenrichment process by using L-tartaric acid, giving the desired compound (1*S*,2*R*)-**1** with a high enantiomeric excess (>99%).

**Scheme 5:**
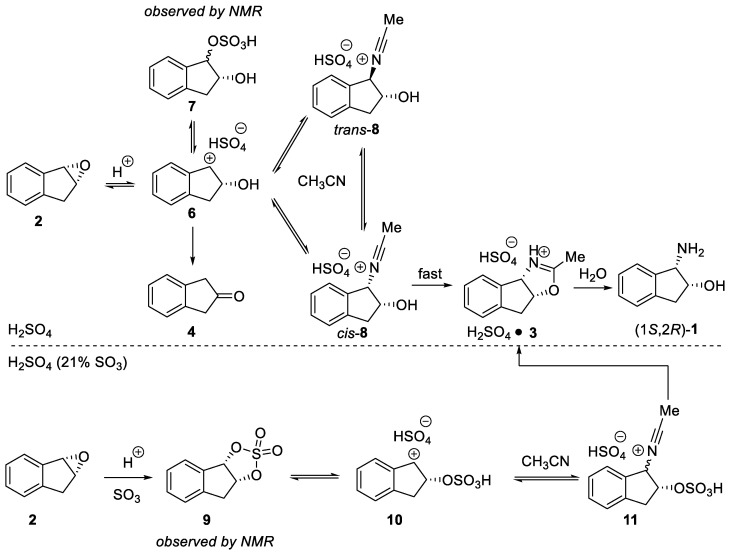
Proposed mechanism for Ritter type reaction from epoxide.

From a mechanistic point of view, the authors proposed that in the presence of H_2_SO_4_ alone, the epoxide **2** is in equilibrium with the opened form **6** (Scheme 5). This carbocation could be reversibly trapped by the counter anion (HSO_4_^−^), giving **7**, which was observed via NMR analysis, or evolve toward the formation of the undesired ketone **4** via 1,2 hydride shift. However, the major pathway consists of its trapping by acetonitrile, leading to the corresponding nitrilium ions *cis*-**8** and *trans*-**8** as a mixture of diastereomers. The success of this reaction is due to the equilibrium between the *cis*- and *trans*-nitrilium **8**, which is displaced by the fast cyclization of the *cis*-nitrilium, leading to **3**. In addition, this mechanism explains the transfer of chiral information from the C_2_ carbon, since no epimerization via the formation of a carbocation occurs at this position. When the reaction is performed in the presence of SO_3_, epoxide **2** is proposed to evolve toward the formation of cyclic sulfate **9** observed at −40 °C via NMR. This intermediate is in equilibrium with the corresponding carbocation **10** through regioselective ring-opening. Suppression of the indanone by-product is likely to result from this fast equilibrium, which probably enables the 1,2 hydride shift process. The trapping of **10** by acetonitrile renders a mixture of *cis* and trans-nitrilium **11**, which cyclizes while releasing SO_3_ to finally deliver the desired *cis*-1-amino-2-indanol with 78–80% yield after hydrolysis.

**Scheme 6:**
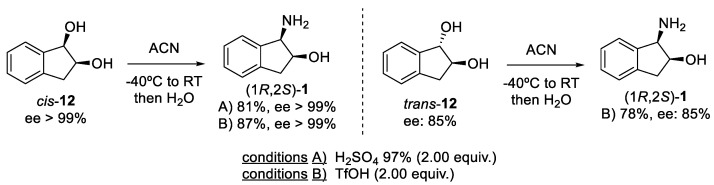
Ritter type reaction from diols.

Interestingly, and in accordance with the mechanism, stereochemistry is controlled by the configuration of the C_2_ center; consequently, the reaction works with enantiopure diol **12** as the starting material (Scheme 6) [14,16]. Indeed, when the reaction was performed with *cis*-1,2 diol or *trans*-1,2 diol, the product was obtained with high yield and without loss of chiral information. It is worthwhile noting that a better yield was obtained when *cis*-diol was used as the substrate instead of epoxide in the presence of H_2_SO_4_ (81% (Scheme 6) vs. 55–60% (Scheme 3)).

To date, numerous methodologies have been developed to obtain enantioenriched epoxide, such as Jacobsen type reactions [17,18], biocatalytic/biomimetic epoxidations [19,20,21] or direct racemic epoxide resolution [22,23]. In addition, epoxide could be obtained from common enantioenriched precursors (i.e., β-hydroxy selenides [24], indene bromohydrin [25]). The enantioselective synthesis of diols **12** is also described in the literature [26,27,28,29,30,31]. It should be underlined that these methodologies—some of which were developed subsequently to the strategies presented here—would most likely be very useful in the preparation of enantiopure *cis*-1-amino-2-indanol.

**Scheme 7:**
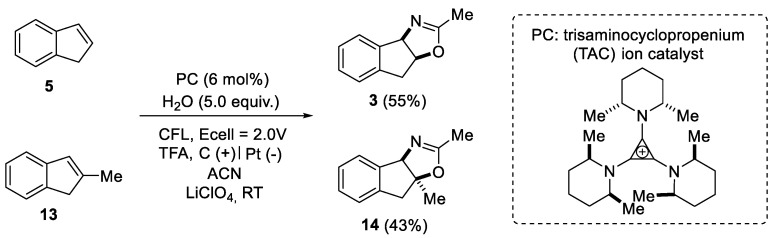
Ritter type reaction from indene under electrophotocatalytic conditions.

More recently, Lambert’s group described an electrophotocatalytic amino-oxygenation reaction of aryl olefins (Scheme 7) [32]. The reaction used a trisaminocyclopropenium (TAC) ion catalyst under electrochemical conditions and compact fluorescent lamp (CFL) irradiation in the presence of acetonitrile and 5.0 equivalent of water. This enabled the formation of oxazolines **3** and **14** with 55% and 43% yield, respectively, from the corresponding indene derivatives.

**Scheme 8:**
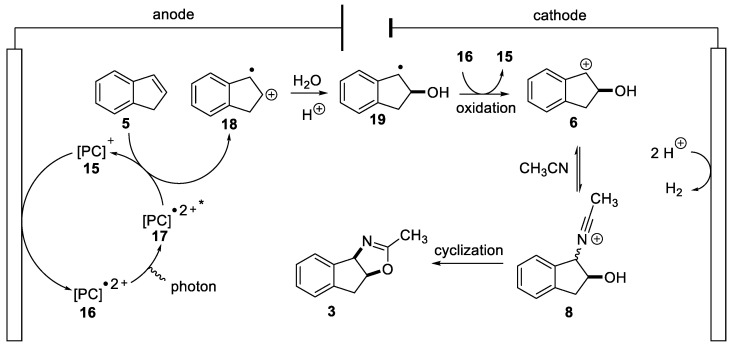
Proposed mechanism for Ritter type reaction from indene under electrophotocatalytic conditions.

Regarding the mechanism (Scheme 8), the authors first proposed an electrochemical oxidation of the photocatalyst **15** to generate the corresponding radical dication **16**. Its photoexcitation renders **17**, which shows high oxidizing properties (E^*^_red_ = 3.3 V vs. SCE) and can thus oxidize indene to furnish the corresponding radical cation **18**. The regioselective trapping of this intermediate by water at C_2_ position leads to benzylic radical intermediate **19**, which in turn could be oxidized by **16** or directly by the anode. This reaction furnishes the benzylic carbocation **6**, which is subsequently reversibly trapped by an equivalent of acetonitrile. The highly favored *cis*-cyclization delivers the final oxazoline **3**. The authors stressed the importance of controlling the amount of water used in the reaction (5.00 equiv.), as a large excess could lead to the trapping of highly electrophilic intermediates generated during the process. Indeed, during the optimization course, when water was used in excess (50.00 equiv.), the authors isolated an aldehyde derivative resulting from probable oxidative cleavage of 1,2 diol intermediates.

#### 2.1.2. Intramolecular Amide Cyclization

Intramolecular amide cyclization is an important class of reaction for the synthesis of *cis*-1-amino-2-indanol. The key point of this strategy is to form an amide/urethane derivative at C_1_ position and a leaving group at C_2_ position, which is engaged in a *O*-amide cyclization step to give the corresponding *cis*-5-membered ring. Two main approaches have been developed to access the key intermediate via either the *N*-opening of epoxide or direct electrophilic activation of indene.

**Scheme 9:**
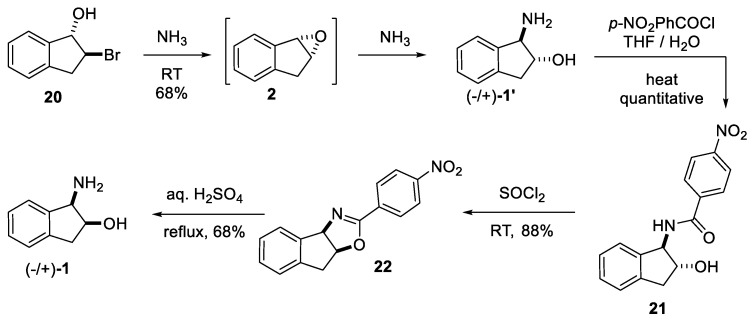
Racemic synthesis of **1** through intramolecular amide cyclization.

This strategy was first reported in 1951 by Lutz and Wayland based on the key formation of a *cis*-oxazoline obtained from the corresponding *trans*-1-amino-2-indanol (−/+)-**1′** (Scheme 9) [33]. The latter was obtained in two steps via addition of ammonia to 2-bromo-1-indanol (**20**), which probably proceeded through an epoxide intermediate to form **1**, followed by quantitative amidation reaction rendering **21**. The addition of thionyl chloride allowed an intramolecular cyclization with inversion of the configuration at C_2_, leading to the *cis*-oxazoline **22**. Finally, acidic hydrolysis at reflux delivered the *cis*-1-amino-2-indanol (−/+)-**1** with 68% yield.

**Scheme 10:**
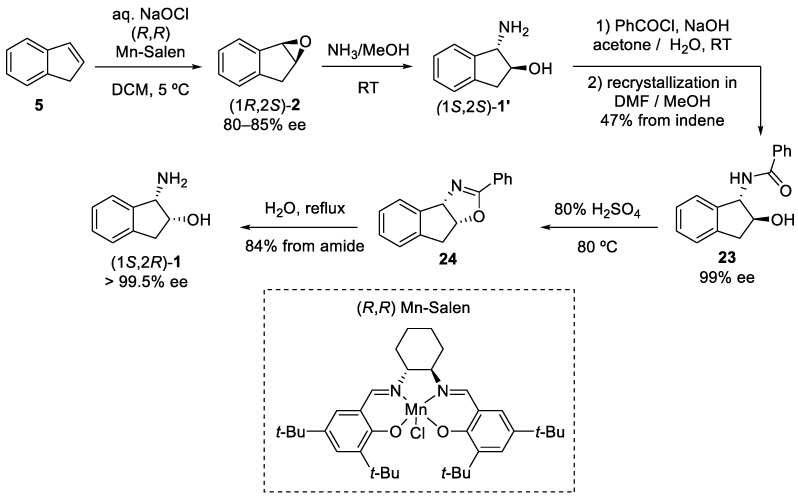
Sepracor synthesis of enantiopure (1*S*,2*R*)-1-amino-2-indanol **1**.

Based on this strategy, researchers from Sepracor developed an enantioselective synthesis of (1*S*,*2R*)-**1** using an enantioselective epoxidation reaction as the key step (Scheme 10) [34]. Indeed, (1*R*,2*S*)-indene oxide (**2**) was prepared in 80–85% ee in the presence of NaOCl and (*R*,*R*)-Mn-Salen. Then, the epoxide was opened with ammonia, giving *trans*-1-amino-2-indanol (**1′**), which subsequently engaged in the amidation reaction under Schotten–Baumann conditions. Recrystallization from a mixture of DMF/MeOH delivered benzamide **23** with 47% yield in three steps from indene **5** and 99% enantiomeric excess. The addition of 80% H_2_SO_4_ at 80 °C to **23** led to the formation of oxazoline **24**, which was directly hydrolyzed, delivering the enantiopure (1*S*,*2R*)-**1** with 84% yield and an enantiomeric excess exceeding 99%.

**Scheme 11:**
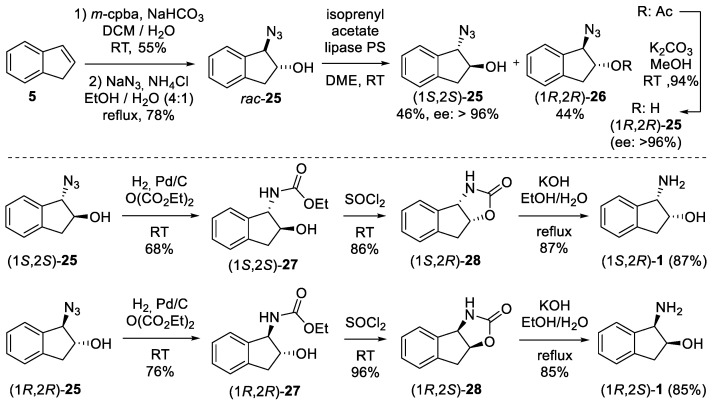
Ghosh synthesis of the two enantiomers of *cis*-1-amino-2-indanol.

In 1997, Ghosh et al. reported the synthesis of both enantiomers of *cis*-1-amino-2-indanol (Scheme 11) [35]. In this approach, racemic *trans*-1-azido-2-indanol (**25**) was obtained in two steps from indene via epoxidation, followed by opening with sodium azide. Enzymatic acylation proceeded in the presence of lipase PS 30 in a mixture of dimethoxyethane (DME) and isopropenyl acetate to give the unreactive alcohol (1*S*,2*S*)-**25** (46% yield, ee > 96%) and acylated enantiomer (1*R*,2*R*)-**26** (44% yield, ee > 96%). Once isolated, both enantiomers were then engaged in the same reaction sequence. The azido group was reduced via hydrogenation and in situ converted into carbamates **27** in the presence of diethylpyrocarbonate. Key cyclization with epimerization of the C_2_ center occurred in SOCl_2_ giving **28** with excellent yields, and a final basic hydrolysis delivered the enantiopure products.

**Scheme 12:**
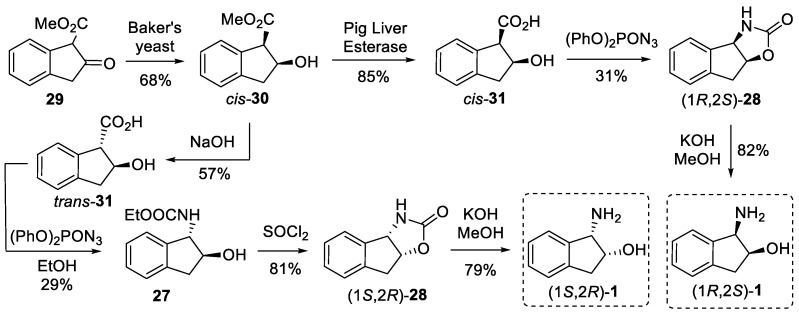
Didier synthesis of the two enantiomers of *cis*-1-amino-2-indanol.

Didier proposed an alternative route for both enantiomers of **1** from β–ketoester **29** (Scheme 12) [36]. Baker’s yeast reduction of **29** yielded the enantiopure 2-hydroxy ester **30**. The hydrolysis of **30** in the presence of NaOH led to a partial racemization at the C_1_ position, providing *trans*-isomer **31** after a selective crystallization. C_1_ epimerization was overcome by enzymatic hydrolysis, leading only to the *cis* isomer **31**. The formation of the C–N bond at the C_1_ position occurred via a Curtius rearrangement triggered by diphenyl phosphorazidate (DPPA), rendering oxazolidinone **28** and carbamate **27** from *cis* and *trans* carboxylic acids, respectively. Basic hydrolysis of **28** led to (1*R*,2*S*)-**1**, whereas its enantiomer (1*S*,2*R*)-**1** was obtained after inversion of the configuration at the C2 position thanks to an intramolecular cyclization of **27** followed by hydrolysis.

**Scheme 13:**
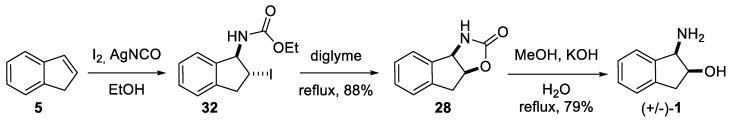
*cis*-1-amino-2-indanol from indene by iodoamidation.

In 1967, Heatchcok et al. proposed a more direct approach through iodoamidation of indene, leading to a *trans* iodocarbamate **32**, which, under reflux in diglyme, yielded the *cis*-oxazolidinone **28** with 88% yield. A final hydrolysis under basic conditions in methanol at reflux afforded the desired racemic compound (−/+)-**1** with 79% yield (Scheme 13) [37].

**Scheme 14:**
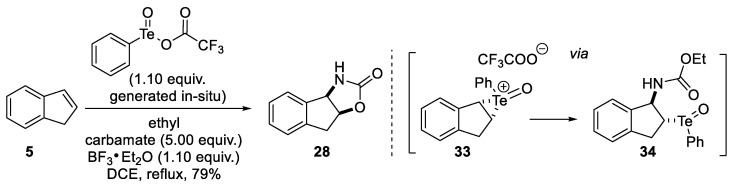
Telluric anhydride-induced formation of oxazolidinone.

In 1989, Ogura and coworkers developed a strategy for accessing oxazolidinone from alkene involving organotelluric compounds under Lewis acid catalysis (Scheme 14) [38,39]. Under the best conditions, indene (**5**) was converted into oxazolidinone **28** with 79% yield in the presence of benzenetellurinyl trifluoroacetate, ethyl carbamate and boron trifluoride etherate under reflux of dichloroethane. In the mechanism proposed by the authors, the activation of the double bond enables its amidotellurinylation via intermediate **33**. Under reflux of dichloroethane and assisted by BF_3_, cyclization of **34** with the inversion at C_2_ center occurred, leading to the corresponding oxazolidinone **28**.

**Scheme 15:**
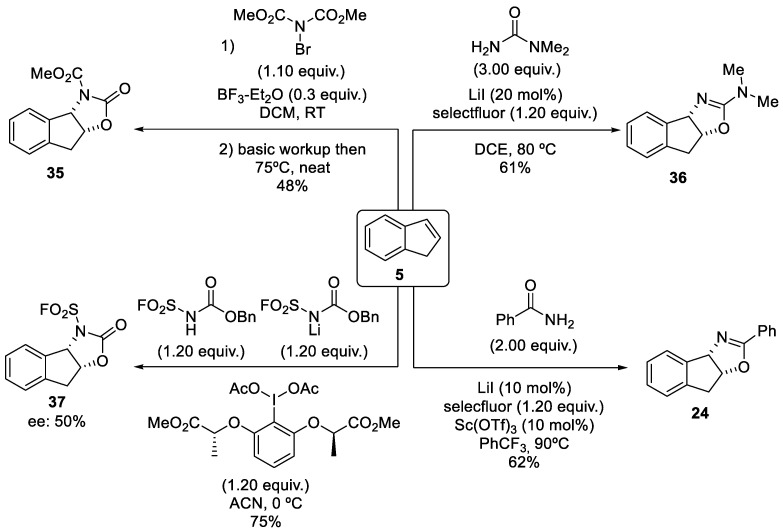
Recent approaches to the amino-oxygenation of indene.

Recently, there has been renewed interest in the amino-oxygenation of alkenes. In 2015, Tepe’s group developed an approach with Br-N-(CO_2_Me)_2_ as the reagent (Scheme 15) [40]. More particularly, indene **5** was transformed into the oxazolidinone **35** precursor of *cis*-1-amino-2-indanol with 48% yield. The authors proposed the conditions for hydrolysis for oxazolidinones (2M LiOH in THF); however, such conditions were not directly applied to **35**. Li and coworkers reported a similar strategy using electrophilic iodine and urea as a partner, rendering *N*-substituted oxazoline **36** [41]. Later, the use of benzamide as a partner was developed to isolate oxazoline **24** with 62% yield [42]. Finally, Hashimoto’s group identified a new carbamate as a bifunctional *N*- and *O*-nucleophile, giving—in the presence of chiral hypervalent iodine—the corresponding oxazolidinone **37** with 75% yield and 50% ee [43].

**Scheme 16:**
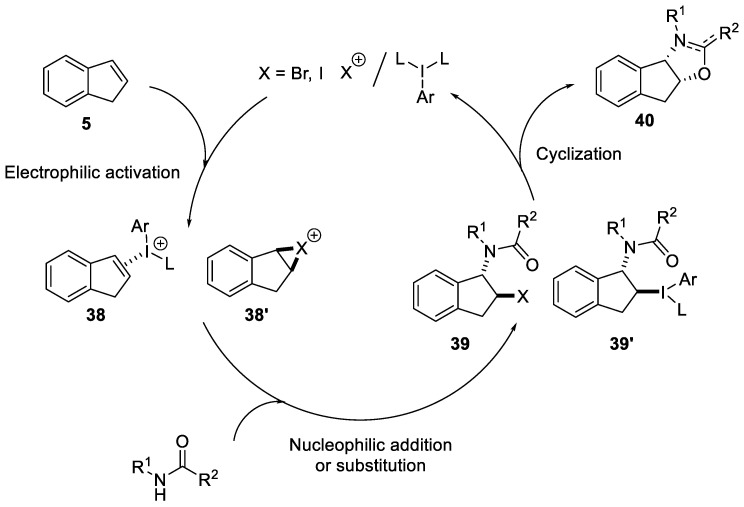
General mechanism of indene amino-oxygenation.

From a mechanistic point of view, this is an electrophilic activation of olefin by bromine—the iodine of hypervalent iodine—leading to intermediates **38** or **38′** (Scheme 16). Then, the *N*-nucleophile reacts with the electrophilic benzylic position of **38** or **38′**, leading to the formation of *trans* amido product **39** or **39′**, followed by an intramolecular cyclization with the nucleophilic oxygen, giving the desired compound **40**.

#### 2.1.3. Benzylic Csp^3^-H Amination

- Benzylic C_sp_^3^-H amination via radical pathway

**Scheme 17:**
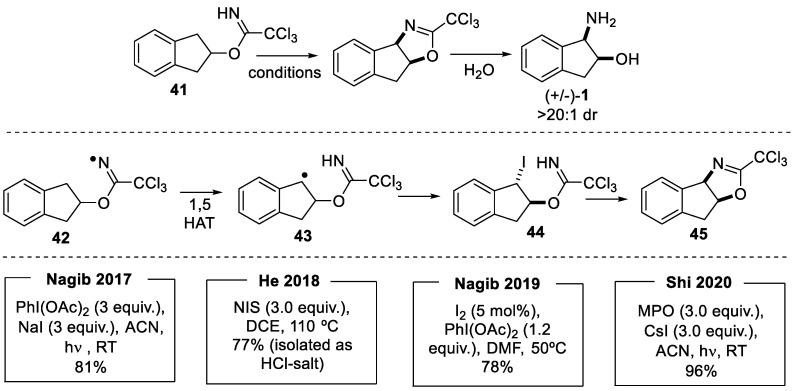
Radical-mediated C–N bond formation.

The use of trichloroacetamidates for radical β-amination reactions for the synthesis of *cis*-1-amino-2-indanol has been proposed by Nagib in 2017 (Scheme 17) [44]. Indeed, when compound **41** was engaged in the reaction in the presence of PhI(OAc)_2_ and NaI under light irradiation in acetonitrile, the desired compound was obtained with a high diastereomeric ratio (>20:1) and 81% yield after hydrolysis of the corresponding oxazoline **45**. The reaction consisted of the formation of an imidate radical 4**2**, which underwent 1,5-hydrogen atom transfer (HAT) to generate the corresponding benzylic radical **43**. The trapping of the carbon-centered radical by iodine led to **44**, followed by intramolecular cyclization. The resulting oxazoline **45** ring was hydrolyzed under acidic conditions, delivering *cis*-1-amino-2-indanol. A similar approach was developed in the meantime by He [45] using three equivalents of NIS in DCE under thermal conditions, and an approach using a catalytic amount of iodine was devised in 2019 by Nagib [46]. Finally, Shi has proposed a new way of obtaining the imidate radical via direct abstraction of the hydrogen of the N–H bond hydrogen atom bond by the malonyl peroxide (MPO) radical [47].

**Scheme 18:**
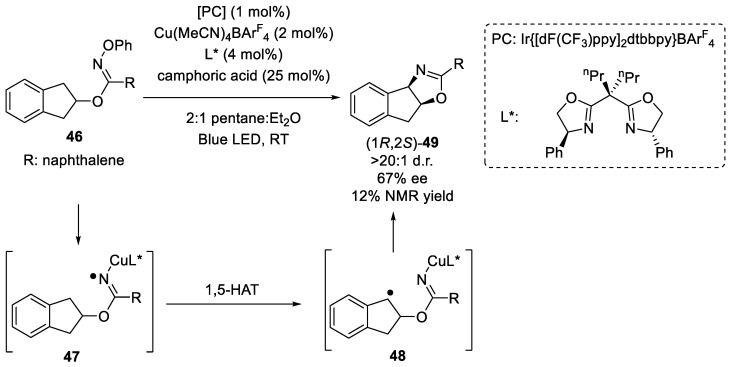
First enantioselective radical-mediated C_1_-N functionalization.

In a very well-designed study on the enantioselective synthesis of β-amino alcohols via radical amination, Nagib’s group used the achiral 2-indanol imidate derivative **46** as a substrate for mechanistic studies (Scheme 18) [48]. When placed under their best conditions, the corresponding oxazoline **49** was obtained with 67% ee and a diastereomeric ratio exceeding 20:1. However, the compound was not isolated, and low yield was obtained (12% NMR yield). The key point in the process is the enantioselective HAT via CuL*-bound imidate **47**, giving the corresponding chiral benzylic radical **48**. It is important to note that the behavior of this substrate seemed to be different to others described in the publication, since better results were obtained. However, this result paved the way for further developments. 

**Scheme 19:**
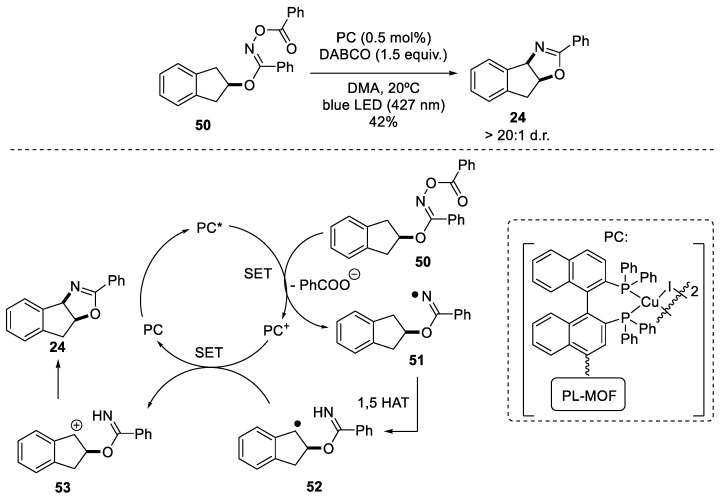
MOF-supported copper-catalyzed oxazoline formation.

Recently, He and coworkers have shown that binap-containing copper photocatalysts on pillar-layered MOF supports were interesting and stable photocatalysts for the conversion of *N*-acyloxy imidates **50** into oxazoline **24** (Scheme 19) [49]. The oxazoline **24** precursor of *cis*-aminoindanol was obtained with 42% yield in the presence of a catalytic amount of the Cu photocatalyst and DABCO in *N*,*N*-dimethylacetamide (DMA) under blue LED irradiation. The reaction starts with the reduction of the *N*-acyloxy imidate **50** by the excited state of the photocatalyst, leading to the formation of a *N*-centered radical **51** and the oxidized photocatalyst (Scheme 19). In the following step, a 1,5 HAT occurs to furnish the benzylic radical **52**, which reacts via single electron transfer (SET) with the oxidized photocatalyst to regenerate it at its ground state. On the other hand, this reaction enables the formation of the corresponding benzylic carbenium ion **53**, which is trapped via intramolecular *N*-cyclization assisted by the base (DABCO).

**Scheme 20:**
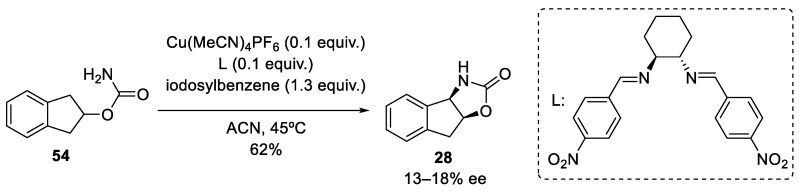
Copper-mediated intramolecular Csp^3^-H amination via radical pathway.

Carbamates **54** derived from 2-indanol are interesting substrates for the synthesis of *cis*-aminoindanol via a radical pathway. Nicholas’ group developed a copper-diimine catalyzed oxidative C-H insertion of carbamates (Scheme 20) [50]. The reaction is proposed to work through the generation of a L-Cu-imido derivative behaving as a triplet-state diradical specie, in which the C-H amination is operated via a stepwise radical process [51]. The authors developed an enantioselective version of this reaction, but moderate enantiomeric excesses were obtained (13–18% ee). The resulting carbamate can be hydrolyzed under several developed conditions [52].

- Benzylic Csp^3^-H amination via nitrene chemistry

**Scheme 21:**
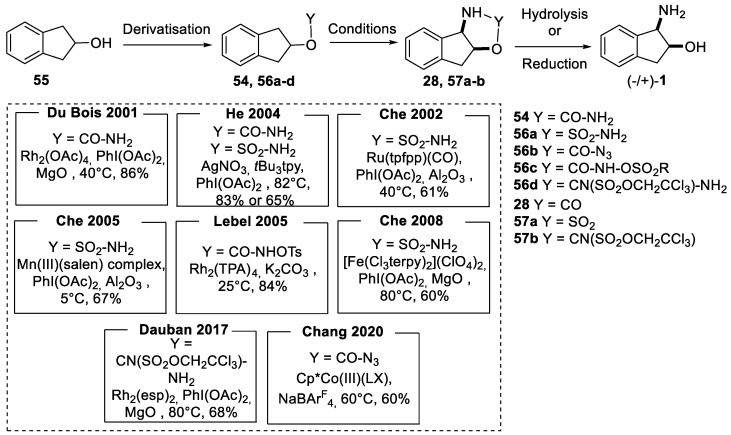
Approaches involving a nitrene intermediate.

2-Indanol **55** is also an attractive substrate for creating the C–N benzylic bond. The amination of the C–H bond occurs from the corresponding primary carbamate **54** [53,54], sulfamate ester **56a** [54,55,56,57,58,59,60], azidoformate **56b** [61], alkylsulfonyloxycarbamate **56c** [62,63,64,65] or carbamimidate **56d** [66] in the presence of various catalysts, such as the complexes of rhodium [53,55,62,63,64,65,66], ruthenium [56,67], silver [54], cobalt [61], manganese [59,60] or iron (Scheme 21) [57,58,59]. The *cis* insertion of the nitrogen atom into the C–H benzylic bond enables the control of stereoselectivity of the reaction thanks to the involvement of a metal-nitrene intermediate. 

**Scheme 22:**
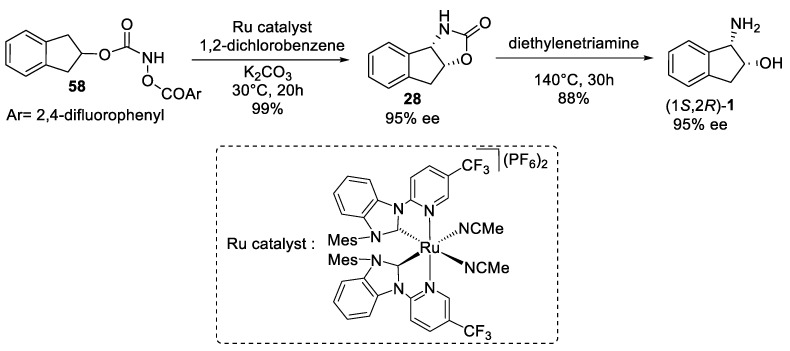
Meggers’ approach to the synthesis of *cis*-1-amino-2-indanol.

The first attempts to desymmetrize carbamate **54** or sulfamate **56a** are reported in the literature. The use of chiral ligands with Rh(II) [68,69], Ru(II) [67] or Mn(III) [60] led to low ee, ranging from 43 to 75%. Recently, Meggers’ group has developed an efficient general approach to chiral oxazolidinone via C(sp^3^)-H nitrene insertion catalyzed with the chiral complexes of Ru(II) associated with *N*-(2-pyridyl)-substituted *N*-heterocyclic carbine (NHC) ligands [70]. In 1,2-dichlorobenzene at 30 °C, the precursor **58** delivered oxazolidinone (3*S*,8*R*)-**28** with 99% yield and 95% ee (Scheme 22).

In all these strategies, the protected amino alcohol must be released from the tricyclic compounds. Hydrolysis of oxazolidinone **28** under the basic condition, typically KOH, led to **1**. For instance, this approach has been widely used in the synthesis of BOX ligands [71,72,73] or biologically active molecules [35]. Recently, new conditions for the hydrolysis of cyclic carbamate in the presence of diethylenetriamine were developed and resulted in **1** with 86% yield [52]. Surprisingly, the conversion of **57a** into (−/+)-**1** was not described in the literature. However, it is well known that the reduction of cyclic sulfatame with LiAlH_4_ yields the corresponding amino alcohol with retention of the configuration [74,75], unlike hydrolysis, which would lead to an inversion of the configuration of the C–O bond and thus to the trans amino-indanol [76]. No example of hydrolysis of a carbamimidate **57b** leading to **1** has yet been reported. However, analogous structures were cleaved under acidic conditions (H_2_SO_4_), releasing the amino-alcohol moiety with good yield [77,78].

### 2.2. cis Stereochemistry Controlled by Epimerization

#### 2.2.1. Epimerization via S_N_2 at C1 Position

**Scheme 23:**
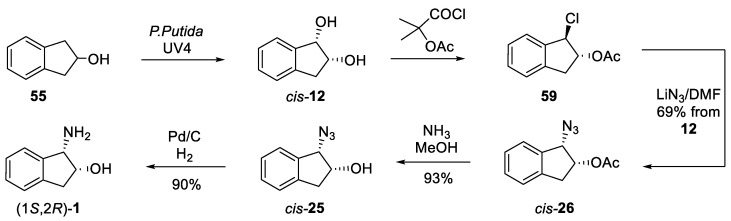
Resnick’s synthesis of *cis*-1-amino-2-indanol.

Resnick’s group proposed a straightforward approach to the synthesis of **1 [79]** from (1*S*,2*R*)-indanediol (**12**, ee 92%) prepared via dioxygenase hydroxylation of 2-indanol **55** (Scheme 23) [80]. The carbon–nitrogen bond in **26** was formed through a two-step sequence, with an overall yield of 69%: (i) concomitant introduction of chlorine in the benzylic position and the acetylation of alcohol in position 2, delivering **59**; and (ii) chlorine substitution by an azide. The aminolysis of **26** followed by the hydrogenation of azide **25** led to (1*S*,2*R*)-**1**. 

#### 2.2.2. Epimerization via Mitsunobu Reaction at C_2_ Position

**Scheme 24:**
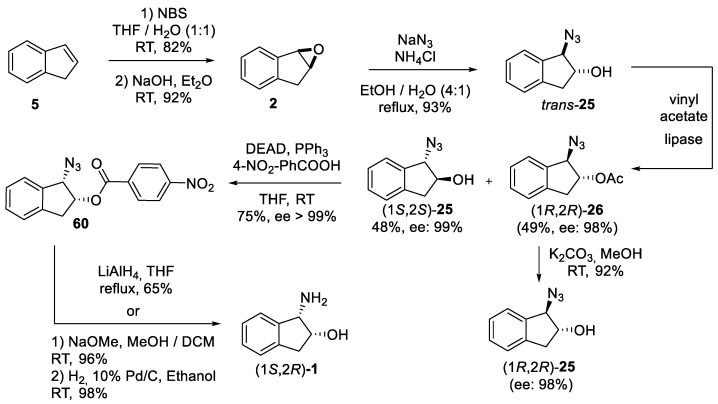
Synthesis of enantiopure *cis*-1-amino-2-indanol via resolution of the corresponding azido-alcohol.

In 1995, Ogasawara and Takahashi reported the synthesis of both enantiomers of *cis*-1-amino-2-indanol via the resolution of *trans*-1-azido-2-indanol (**25**) (Scheme 24) [81]. The racemic epoxide **2** was obtained in a two-step sequence via treatment of indene **5** with NBS to render trans-bromohydrin with 82% yield, followed by an epoxidation step mediated by NaOH. The opening of the epoxide by sodium azide rendered the racemic *trans*-1-azido-2-indanol (**25**) with 93% yield. A screening of the conditions allowed the authors to identify Lipase PS (*Pseudomonas* sp. Amano) and vinyl acetate in *tert*-butyl methyl ether as the best conditions for alcohol resolution. Unreacted *trans*-(1*S*,2*S*)-azido-2-indanol (**25**) was recovered with 48% yield and 99% ee, whereas azido acetate (1*R*,2*R*)-**26** was obtained with 49% yield and 98% ee. The latter was then converted into the corresponding alcohol via methanolysis with 92% yield without the erosion of chirality. On the other hand, Mitsunobu inversion of **25** was carried out in the presence of *p*-nitrobenzoic acid, diethyl azodicarboxylate (DEAD) and triphenylphosphine, rendering the corresponding *cis*-azido ester **60** with 75% yield. At this stage, two pathways were proposed to reach the enantiopure *cis*-1-amino-2-indanol (1*S*,2*R*)-**1**, either via concomitant reduction of the azido group and the ester with LiAlH_4_ in THF (65% yield) or through a two-step sequence consisting of methanolysis of the ester followed by hydrogenation over palladium on carbon (98% yield). The same strategy was successfully applied to transform the other trans-azido-alcohol enantiomer into (1*R*,2*S*)-**1**.

### 2.3. cis Stereochemistry Controlled by Diastereoselective Imino Alcohol Reduction

**Scheme 25:**
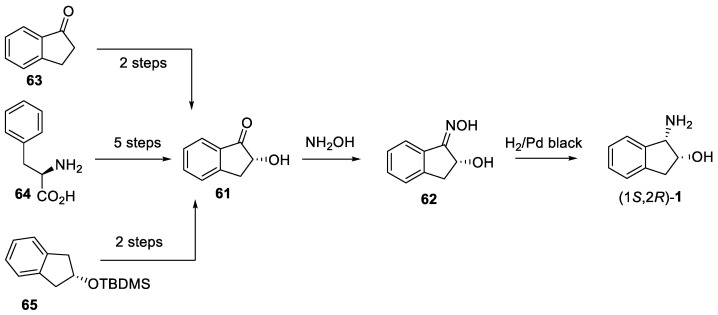
Synthesis of (1*S*,2*R*)-**1** from 2-hydroxy-1-indanone.

In the 1-amino-2-indanol **1** route, 2-hydroxy-1-indanone (**61**) turned out to be an interesting intermediate (Scheme 25). Different strategies were proposed for its preparation in its enantiomerically pure form. Using the chiral pool, (*R*)-phenylalanine (**64**) was converted in five steps to (*R*)-**61** [82]. Alternatively, (*R*)-**61** was also synthesized in two steps from 1-indanone (**63**) thanks to an α acetoxylation of the ketone, followed by enzymatic resolution [82,83]. Desymmetrization of protected 2-indanol **65** through the oxidation of the benzylic position with Mn(salen) complexes also yielded (*R*)-**61** with a modest yield (13%, 70% ee) [84]. The conversion of (*R*)-**2** into (1*S*, 2*R*)-**1** was performed in two steps: (i) formation of the oxime; and (ii) diastereoselective reduction with H_2_/Pd [82,84] or in the presence of oxazaborolidine complexes prepared from different chiral amino alcohols and BH_3_ [83].

**Scheme 26:**
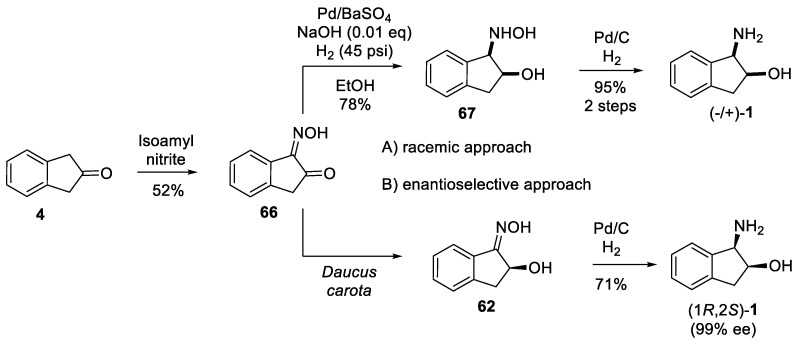
Synthesis of **1** from 1,2-indanedion-1-oxime.

Direct reduction of 1,2-indanedion-1-oxime (**66**)—prepared from 2-indanone (**4**) via oximination—with H_2_ in the presence of Pd/BaSO_4_ led to the corresponding racemic *cis*-*N*-hydroxyamine **67** (Scheme 26A) [85]. Subsequent hydrogenation of the *N*-hydroxyamine moiety enabled (−/+)-**1**. 

An analogous enantioselective approach was developed with sequential reductions (Scheme 26B). The first reduction with a biocatalyst—*Daucus carota*—led to alcohol **62**, and then, the oxime function was hydrogenated to an amino group with H_2_ in the presence of Pd/C with 99% ee and 95% conversion in two steps [86].

## 3. Syntheses from Non-Indane Skeleton

### 3.1. Synthesis from (E)-Cinnamate Ethyl Ester

**Scheme 27:**
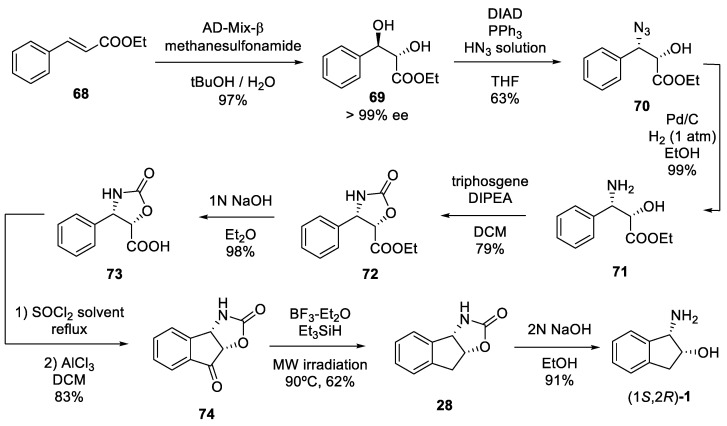
Ko’s synthesis of *cis*-1-amino-2-indanol.

In 2006, Ko’s group reported an eight-step enantioselective synthesis of *cis*-1-amino-2-indanol from (*E*)-cinnamate ethyl ester (**68**) (Scheme 27) [87]. The synthesis started with a Sharpless asymmetric dihydroxylation, leading to the corresponding *syn*-diol **69** with 97% yield and 99% ee. The benzylic alcohol was then selectively substituted with inversion of the configuration under Mitsunobu conditions using HN_3_ as a nucleophile delivering **70** [88]. After reduction of the azide **70** under a hydrogen atmosphere, the corresponding amino alcohol **71** was judiciously protected with 79% yield under oxazolidinone form **72** in the presence of triphosgene, allowing the protection and further deprotection of both alcohol and amine in a single step. The ethyl ester **72** is then engaged in a saponification reaction, in which the co-solvent (Et_2_O) plays an important role. Indeed, the choice of a more polar solvent, such as THF, leads to detectable epimerization via ^1^H NMR. The indane ring skeleton was introduced with 83% yield via a Friedel–Crafts acylation, first converting the carboxylic acid **73** into its acyl chloride derivative and then via addition of an excess of AlCl_3_. The corresponding indanone **74** was then reduced by an excess of silane catalyzed by BF_3_^●^Et_2_O under microwave irradiation, since low conversions were observed under thermal conditions (oil bath). The *cis*-1-amino-2-indanol was obtained after final deprotection of the oxazolidinone ring **28** under basic conditions. 

### 3.2. Synthesis from 7,3-Xylofuranose Derivative

**Scheme 28:**
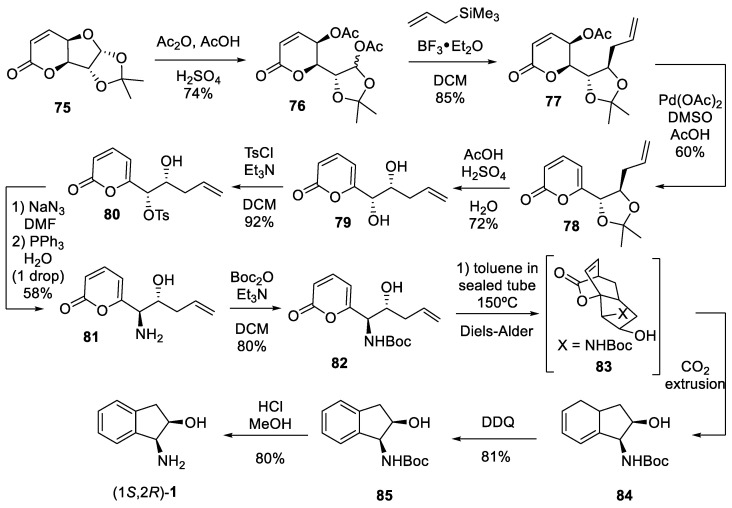
Synthesis of *cis*-1-amino-2-indanol from 7,3-xylofuranose derivative.

In 2021, a successful enantioselective synthesis of *cis*-1-amino-2-indanol using the Diels–Alder reaction as a key step was reported (Scheme 28) [89]. The chiral pool employed for the synthesis was the versatile chiron 7,3-xylofuranose derivative (7,3-LXF) prepared in two steps from diacetone-D-glucose [90]. 7,3-LXF was first transformed into diacetylated derivative **76** under acidic conditions. Then, diastereomeric allylation was achieved with 85% yield in the presence of allylsilane and BF_3_^●^Et_2_O, followed by Pd-catalyzed β-elimination, rendering **78** with 60% yield. Acidic deprotection allowed the formation of diol **79**, followed by a selective tosylation reaction, enabling the introduction—in the second sequence—of the amine group with an appropriate configuration. In order to avoid the formation of by-products during the Diels–Alder step, the amine **81** was first protected to render **82** and then refluxed in toluene at 150 °C, rendering—after CO_2_ extrusion from **83**—compound **84**. Rearomatization via DDQ led to **85** with 81% yield, and the final removal of the Boc group under acidic conditions afforded the desired *cis*-1-amino-2-indanol with 80% yield.

### 3.3. Synthesis from D-Phenylalanine

**Scheme 29:**
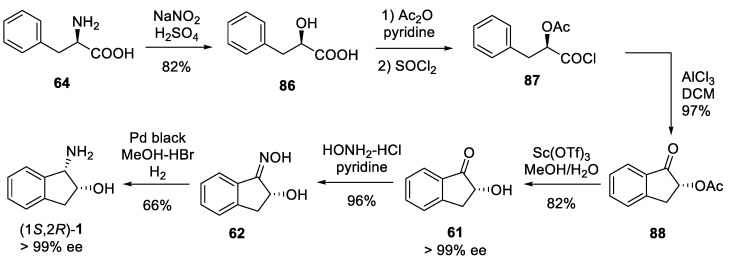
Synthesis of *cis*-1-amino-2-indanol from D-phenylalanine.

α-amino acids such as phenylalanine provide a readily available chiral pool for synthesis. Hiyama and coworkers reported a synthesis of enantiomerically pure (1*S*,2*R*)-1-amino-2-indanol (**1**), starting from phenylalanine (Scheme 29) [91]. The reaction began by the conversion of D-phenylalanine (**64**) into the corresponding optically pure hydroxylated compound **86** with 82% yield using a mixture of NaNO_2_–H_2_SO_4_ [92]. The latter was then protected and the carboxylic acid transformed in acyl chloride with thionyl chloride at 50 °C. The addition of AlCl_3_ to **87** led to the cyclic structure **88** via Friedel–Crafts acylation without the loss of chiral information. A screening of several conditions for the hydrolysis of **88** without epimerization enabled the identification of the best conditions, i.e., Sc(OTf)_3_ (20 mol%) in a mixture of H_2_O–MeOH (1:4) at room temperature, rendering **61** with 82% yield and >99% ee. The α-hydroxy ketone **61** was transformed into its oxime equivalent **62** obtained as a mixture of isomers. After optimization, the authors found out that diastereoselective hydrogenation using Pd black in MeOH/HBr afforded the final product (1*S*,2*R*)-**1** with 66% yield.

## 4. Resolution

As previously mentioned, enantiopure *cis*-1-amino-2-indanol can be prepared from an enantiopure starting material, such as an epoxide, a diol or an azido-alcohol. However, a significant number of syntheses provide a racemic form of *cis*-1-amino-2-indanol. Various approaches have been proposed for resolving this racemic mixture.

### 4.1. Chemical Resolution

**Scheme 30:**
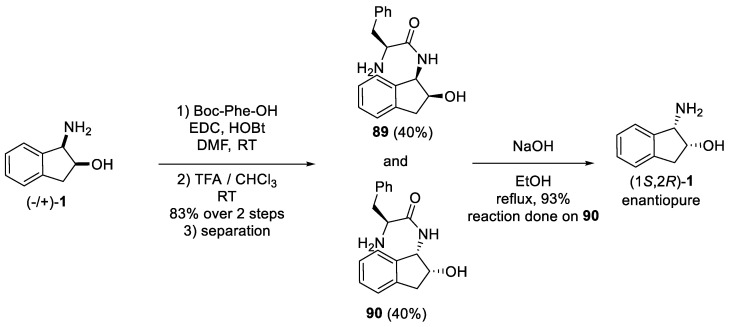
Chemical resolution of racemic *cis*-1-amino-2-indanol.

Interestingly, the resolution of racemic *cis*-1-amino-2-indanol was performed after its derivatization and chromatographic separation (Scheme 30) [93]. After the preparation of racemic *cis*-1-amino-2-indanol, following the Lutz and Wayland strategy, the authors performed peptide coupling with Boc-Phe-OH followed by Boc deprotection, leading to the formation of a pair of diastereomers **89** and **90** separable via chromatography on silica gel. Both diastereomers were isolated, each with 40% yield, and enantioenriched *cis*-1-amino-2-indanol was isolated with 93% yield after cleavage of the amide bond.

**Scheme 31:**
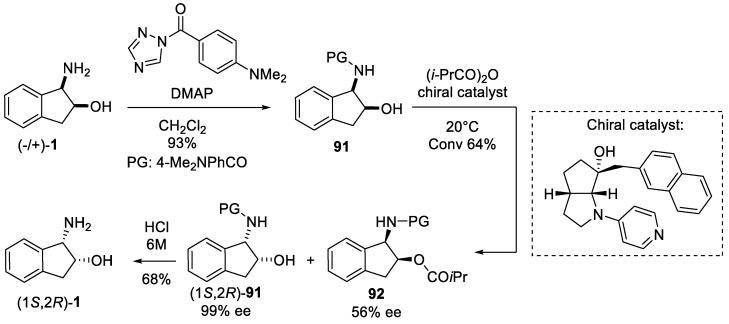
Kawabata’s kinetic resolution via enantioselective acylation.

Another general strategy for β-amino-alcohol resolution is to perform a kinetic resolution via enantioselective acylation of the alcohol in the presence of a chiral nucleophilic catalyst. Kawabata’s group implemented this approach in *cis*-1-amino-2-indanol with a chiral aminopyridine as a catalyst (Scheme 31) [94]. After a screening, 4-(dimethylamino)benzamide was determined to be the best protecting group for the amine function. In the presence of (*i*-PrCO)_2_O as the acylating agent and the chiral amino pyridine, the *S* alcohol from mixture **91** was preferentially acylated to **92**, and the unconverted protected β-amido-alcohol **(**1*S*,2*R*)-**91** could be recovered with an 99% ee after 64% conversion. A treatment with 6M HCl provided the targeted (1*S*,2*R*)-1-amino-2-indanol **1** with 68% yield. An analogous approach was proposed by Campbell with a *N*-4′-pyridinyl-α-methyl proline derivative as the catalyst and trifluoroacetyl as the nitrogen protecting group in order to facilitate its removal [95].

**Scheme 32:**
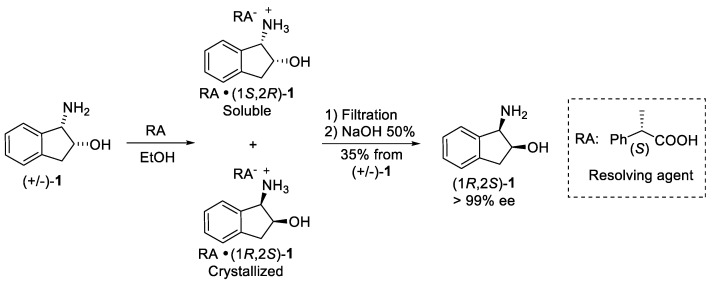
Resolution of *cis*-1-amino-2-indanol via diastereomeric salt formation.

Resolution of the racemic *cis*-1-amino-2-indanol can also be achieved via diastereomeric salt formation (Scheme 32). (*S*)-2-Phenylpropionic acid proved to be an efficient resolving agent, inducing a selective crystallization of the ammonium salt formed with (1*R*,2*S*)-1-amino-2-indanol (**1**). After filtration, the amino alcohol could be released under a basic work-up, leading to an enantiopure product with 35% yield, and the resolving agent recovered with 93% yield [96]. Similar results were obtained using tartaric acid as a resolving agent, either to perform enantioenrichment [15] or complete resolution [97].

### 4.2. Enzymatic Resolution

**Scheme 33:**
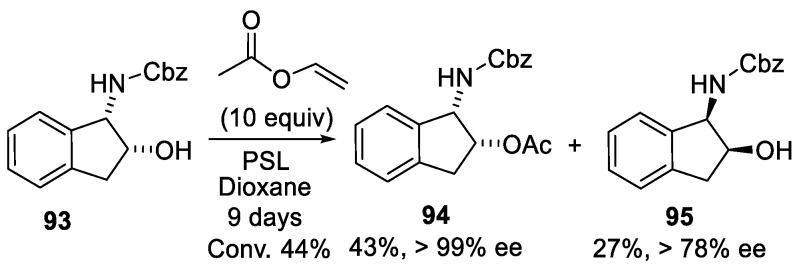
*R* selective enzymatic acylation of *N*-Cbz protected racemic *cis*-1-amino-2-indanol.

Enzymatic resolution is an interesting alternative. Various approaches have been devised involving the reactivity of alcohol or amine functions. Gotor’s group proposed the acylation of *N*-Cbz protected racemic *cis*-1-amino-2-indanol **93** with vinyl acetate catalyzed by *Pseudomonas cepacia* lipase (PSL) (Scheme 33). These conditions enabled a *R* selective acylation of the alcohol **93** (43%, >99% ee) with 44% conversion [98].

**Scheme 34:**
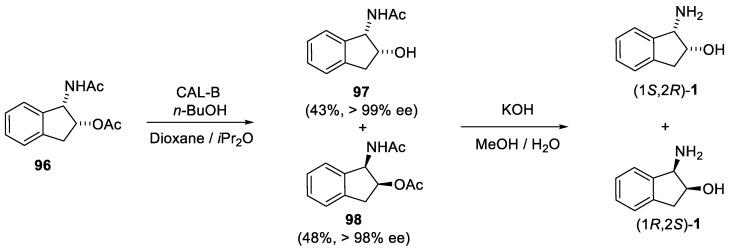
*R* selective enzymatic hydrolysis of *N*,*O*-Ac protected racemic *cis*-1-amino-2-indanol.

The enzyme’s ability to hydrolyze an ester was tested on *N*,*O*-diacetyl-*cis*-1-amino-2-indanol **96** (Scheme 34) [99]. The alcoholysis of **96** was carried out in the presence of *Candida antartica* lipase B (CAL-B) and *n*-butanol. The very high enantiomeric ratio (E > 500) provided both the hydrolyzed ester **97** as well as the unreactive ester **98** with excellent ee and yields. The targeted amino-indanol was released under basic conditions without eroding the ee.

**Scheme 35:**
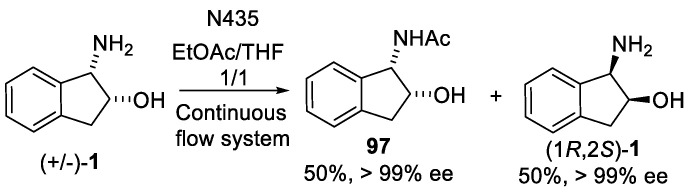
Continuous-flow kinetic resolution of *cis*-1-amino-2-indanol with Novozym 435.

Interestingly, more recently, a continuous-flow resolution was directly performed on *cis*-1-amino-2-indanol with CAL-B immobilized on an acrylic resin (Novozym 435^®^ (N435)) with EtOAc as the acyl donor (Scheme 35) [100]. The flow system enabled selective acylation of the amino group of the (1*S*,2*R*) substrate, whereas analogous conditions in vials were much less effective, certainly because the controlled flow rate increased the local concentration of immobilized CAL-B, and consequently, the amino group acylation rate. The result was a conversion of 50% and an ee of 99% for the two alcohols recovered with E > 200.

## 5. Substituted *cis*-1-Amino-2-Indanol

Due to the high interest in them for catalysis and for the development of drug candidates, numerous *cis*-1-amino-2-indanol derivatives with a substituent on the aromatic ring have been prepared. Two different strategies are implemented: (i) a post-functionalization approach, in which the *cis*-1-amino-2-indanol moiety is already formed, and substituents are introduced via coupling reactions; or (ii) a pre-functionalization approach, in which the introduction of the substituent to the aromatic ring is performed before the formation of the *cis*-1-amino-2-indanol skeleton with the strategies described above. This section presents an overview of the structure of substituted *cis*-1-amino-2-indanols and their preparation strategy.

### 5.1. Post-Functionalization

**Scheme 36:**
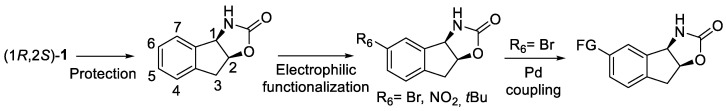
General strategy of post-functionalization.

The post-functionalization strategy has been used in particular to prepare new catalysts based on *cis*-1-amino-2-indanol architecture (Scheme 36). This strategy offers the advantage of utilizing the commercially available enantiopure *cis*-1-amino-2-indanol, which is first protected under the carbamate form. At this stage, functionalization at position 6 can be performed through an electrophilic reaction, such as bromination [71,72,101], nitration [102] or Friedel–Crafts reaction [71]. In addition to the Friedel–Crafts reaction, the C–C bond can be formed in two steps via a metal-catalyzed coupling reaction on the corresponding bromide [71,72,73,101]; an alkyl or aryl substituent can be introduced in this manner (Table 1). It should be pointed out that the regioselectivity of the electrophilic reaction mainly leads to substitution at position 6 (R_6_), which limits the scope of this approach.

### 5.2. Pre-Functionalization

**Scheme 37:**
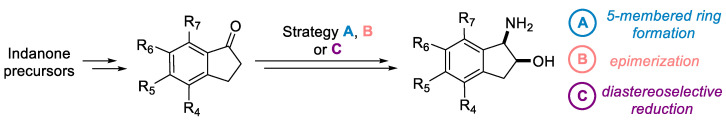
General strategy of pre-functionalization.

The pre-functionalization approach implements the various strategies discussed earlier in this review, namely the Ritter reaction (Strategy A, Scheme 2), the Mitsunobu reaction (Strategy B, Scheme 2) or a reductive approach (Strategy C, Scheme 2). The difference lies in the starting substrate, indanone, which already carries substituents on the aromatic ring (Scheme 37). It offers the possibility of choosing different regioisomers, as well as poly-substituted products (Table 2). However, depending on the regioisomer desired, the scope of the reaction is limited. For example, at position 7 (R_7_), only alkyl groups were described (entries 1 and 2) [86,104,105,106,107]. Regarding position 6 (R_6_), a larger variety of electron-donating or electron-withdrawing substituents (fluorine [108], bromine [109], methoxy [110,111] and nitro [112]) were introduced onto indanone prior to conversion, mainly via a Ritter-type reaction (entries 3–6). After the formation of *cis*-1-amino-2-indanol, the nitro group can be easily reduced to an amino group [112]. The introduction of fluorine [108], chlorine [113] or bromine [109] using this strategy is also possible at position 5 (R_5_) (entries 7–9). It may be underlined that the presence of bromine in position 5, which is not possible using the post-functionalization approach, opens the way to subsequent functionalization. Substitution at position 4 (R_4_) seems to be more difficult (or less interesting) to achieve, since only methyl (entry 10) [86,106] and fluoro (entry 11) [114] derivatives were prepared. Di-bromo-substituted [86] and polyaromatic indanones [115,116,117,118] were also used for the preparation of *cis*-1-amino-2-indanol moieties (entries 12–14).

## 6. Conclusions

*cis*-1-amino-2-indanol is a ubiquitous substructure in many areas of chemistry, and its synthesis has attracted the attention of many chemists in both academic and industrial fields for decades. A wide range of strategies have been implemented to prepare this molecule in order to overcome its main structural feature, namely the *cis*-introduction of an amino group and an alcohol group in positions 1 and 2, respectively. Most of the time, stereochemistry is controlled by forming a 5-membered intermediate ring. In this context, indene has proven to be a versatile starting material, either as a precursor of key functional groups, such as epoxide, diol, halohydrin in their racemic or enantiopure form, or directly functionalized via electrophilic activation of its double bond. Among the implemented strategies, direct C-H functionalization has recently attracted a great deal of interest. These promising approaches involve nitrene or radical intermediates. However, such strategies are mainly developed in their racemic versions, and the few attempts of enantioselective approaches have yielded low enantiomeric excesses. Moreover, the scope of all reactions is particularly limited in terms of the diversity of substitution on the aromatic ring. Position 6 offers the largest possibility of substitution by an electron-withdrawing or electron-donating group. The examples for positions 4, 5 and 7 are scarce and only concern alkyl or halogen groups. No electron-rich or electron-poor groups have been introduced on these positions, and consequently, their impact on the methods implemented for the introduction of the amino-alcohol moiety—in the pre-functionalization strategy—remains unknown. In this context, the development of functionalizations through Csp^3^-H activation provides interesting alternatives, as the reaction intermediates involved are different from those involved in older synthetic approaches.

## Figures and Tables

**Table 1:** Molecules obtained via the post-functionalization strategy.

Entry	R_4_	R_5_	R_6_	R_7_	Strategy
1	H	H	Br	H	Bromination [71,72,101,102]
2	H	H	*t*Bu	H	Friedel–Crafts [71]
3	H	H	Cy	H	Bromination/Coupling [71,72,101]
4	H	H	NO_2_	H	Nitration [103]
5	H	H	Ar and derivatives	H	Bromination/Coupling [72,101,102]
6	H	H	*i*Pr	H	Bromination/Coupling [72,73]
7	H	Ad	Ad	H	Bromination/Coupling [72]

**Table 2:** Molecules obtained via the pre-functionalization strategy.

Entry	R_4_	R_5_	R_6_	R_7_	Strategy
1	H	H	H	Me	Strategy A—Ritter [104,105,106] Strategy C—Reduction [86]
2	H	H	H	*i*Pr	Strategy A—Ritter [105,106,107] Strategy C—Reduction [86]
3	H	H	F	H	Strategy A—Ritter [108]
4	H	H	Br	H	Strategy A—Ritter [109]
5	H	H	OMe	H	Strategy A—Ritter [110] Strategy B—Mitsunobu [111]
6	H	H	NO_2_	H	Strategy A—Ritter [112]
7	H	Cl	H	H	Strategy A—Ritter [113]
8	H	F	H	H	Strategy A—Ritter [108]
9	H	Br	H	H	Strategy C—Reduction [86,119]
10	Me	H	H	H	Strategy A—Ritter [106] Strategy C—Reduction [86]
11	F	H	H	H	Strategy C—Reduction [114]
12	H	Br	Br	H	Strategy C—Reduction [86]
13	H	Π-extended (biaryl)		H	Strategy A—Ritter [115,116] Strategy B—Mitsunobu [117]
14	H	H	Π-extended (biaryl)		Strategy A—Ritter [118]

## Data Availability

Not applicable.
